# Armament Imbalances: Match and Mismatch in Plant-Pollinator Traits of Highly Specialized Long-Spurred Orchids

**DOI:** 10.1371/journal.pone.0041878

**Published:** 2012-07-25

**Authors:** Marcela Moré, Felipe W. Amorim, Santiago Benitez-Vieyra, A. Martin Medina, Marlies Sazima, Andrea A. Cocucci

**Affiliations:** 1 Laboratorio de Ecología Evolutiva y Biología Floral, Instituto Multidisciplinario de Biología Vegetal, Consejo Nacional de Investigaciones Científicas y Técnicas - Universidad Nacional de Córdoba, Córdoba, Argentina; 2 Programa de Pós-Graduação em Biologia Vegetal, Departamento de Biologia Vegetal, Instituto de Biologia, Universidade Estadual de Campinas, Campinas, São Paulo, Brasil; 3 Departamento de Biologia Vegetal, Instituto de Biologia, Universidade Estadual de Campinas, Campinas, São Paulo, Brasil; Centro de Investigación y de Estudios Avanzados, Mexico

## Abstract

**Background:**

Some species of long-spurred orchids achieve pollination by a close association with long-tongued hawkmoths. Among them, several *Habenaria* species present specialized mechanisms, where pollination success depends on the attachment of pollinaria onto the heads of hawkmoths with very long proboscises. However, in the Neotropical region such moths are less abundant than their shorter-tongued relatives and are also prone to population fluctuations. Both factors may give rise to differences in pollinator-mediated selection on floral traits through time and space.

**Methodology/Principal Findings:**

We characterized hawkmoth assemblages and estimated phenotypic selection gradients on orchid spur lengths in populations of three South American *Habenaria* species. We examined the match between hawkmoth proboscis and flower spur lengths to determine whether pollinators may act as selective agents on flower morphology. We found significant directional selection on spur length only in *Habenaria gourlieana*, where most pollinators had proboscises longer than the mean of orchid spur length.

**Conclusions/Significance:**

Phenotypic selection is dependent on the mutual match between pollinator and flower morphologies. However, our findings indicate that pollinator-mediated selection may vary through time and space according to local variations in pollinator assemblages.

## Introduction

One of the most important aims in the study of floral evolution is to evaluate the role that pollinators play in moulding flower form [1]-[4]. This issue acquires particular relevance in the case of plants pollinated by long-tongued hawkmoths where a mutual matching between proboscis and flower lengths is necessary for successful pollination [1], [5], [6]. This system was first pointed out by Darwin [1] to exemplify the mechanism of a coevolutionary race, in which hawkmoth proboscis length and nectar spur length were expected to reciprocally act as selective agents constantly and gradually driving a positive shift in adaptive peaks [7].

Long-tongued hawkmoths have an advantage in terms of nectar intake because they can access a broader range of flower corolla lengths than short-tongued individuals, and so, these hawkmoths are released from the level of competition experienced by small- and modal-tongued individuals [8]. Similarly, long flowers have an advantage over short ones, because they benefit from an increase in pollination effectiveness through improved contact between flower and pollinator. Furthermore, if long-tongued hawkmoths reject shorter flowers because long flowers provide higher energetic rewards, long flowers are likely to benefit from an increase of pollinator quality and quantity [8]. Thus, spurs longer than moths’ proboscises are positively selected up to some critical point where the nectar becomes inaccessible to the moths and pollinator-mediated selection becomes stabilizing [8], [9]. In addition to this paired coevolutionary scenario, two alternative hypotheses that take into account community aspects have been proposed: the pollinator shift model, which posits a punctuated evolution of spur length due to a switch to new pollinators with longer tongues [10], [11], and the optimal foraging model [12]-[14,], where the foraging strategy of pollinators triggers the coevolution of long proboscises and deep corolla tubes. Moreover, at a larger temporal and spatial scale (e.g. the Neotropics), it has been proposed that the mechanism behind spur and proboscis elongation is one of diffuse evolutionary interactions among plants and pollinators that generate and maintain general trends in space and time [8].

Natural selection driven by pollinators may modify the distribution of one or more floral traits within a population, particularly those associated with the precise functioning of the pollination mechanism [15]-[17]. These traits are expected to be subject to directional [15], [18]-[22] or (once the optimum is achieved) stabilizing selection [23], [24]. These expectations presume that pollinator mouthparts are equal or longer than the floral tube length. However, mismatches may occur due to spatio-temporal fluctuations in pollinator assemblages [25]. Hence, long-tongued pollinators, those capable to reach hidden nectar in extremely deep flower tubes, may be unreliable pollinators across time and space [26].

Hawkmoth assemblages in the Neotropical region consist mainly of short-tongued species that are more abundant than their few long-tongued counterparts [8], [27]-[31]. Since pollination of long-spurred species is highly dependent on these less abundant hawkmoths, we would expect marked variation in their reproductive success according to spatio-temporal differences in the pollinator assemblages. Moreover, stabilizing or positive directional patterns of pollinator-mediated phenotypic selection may only take place when the hawkmoth tongue lengths equal or exceed the average of the spur length in a plant population. Consequently, populations of extremely long-spurred orchids offer a model system to test these expectations on selection patterns.

Although the relationships between hawkmoths and orchids have been used as a classical model for studies on flower evolution [11], [15], [16], [18], no studies on pollinator-mediated selection have addressed this subject in orchids with extremely long floral tubes. In this context, the aim of this study was to analyse the occurrence of pollinator-mediated selection in long-spurred South American orchids. We surveyed the hawkmoth faunas in a Montane Grassland area in Central Argentina and in a Montane Atlantic Rainforest area in Southeastern Brazil, and we analyzed the match of tongue and spur lengths distributions to address whether pollinators may act as selective agents on flower morphology. We also investigated the phenotypic selection patterns in *Habenaria* species occurring in these areas: *Habenaria gourlieana* Gillies ex Lindl., *Habenaria johannensis* Barb. Rodr. and *Habenaria paulistana* J. A. N. Bat. & Bianch. These three *Habenaria* species have very precise pollination mechanisms in which the pollinaria are attached to the heads of long-tongued hawkmoths due to a mechanical fit between the flower and pollinator morphologies (F.W. Amorim, G.E. Wyatt, M. Sazima unpublished data) [32].

## Methods

### Ethics Statement

All necessary permits were obtained for the described field studies. Instituto Florestal of the São Paulo State Environment Department provided the grant permission (process n°: 000.401/2008) for field work in the Núcleo Santa Virgínia at Serra do Mar State Park, Brazil, and the owners provided permission for field work in El Durazno, Argentina. No field studies involved endangered or protected species.

### Study System

**Figure 1:**
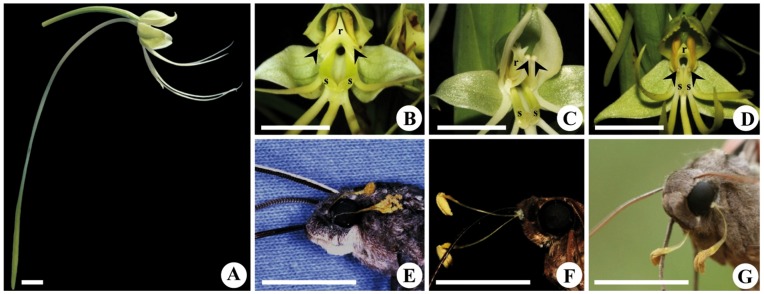
Flower morphology of three South American *Habenaria* species and place of pollinaria attachment onto the pollinator’s head. A) Lateral view of *H. gourlieana* flower depicting the general morphology of the three long-spurred *Habenaria* species studied. Detail of the rostellum morphology (r), position of the viscidia (arrow heads) and stigmatic surfaces (s) in B) *H. gourlieana*; C) *H. johannensis* and D) *H. paulistana.* E) *H. gourlieana* pollinaria attached to the fore margin of *Manduca sexta* eye. F) *H. johannensis* pollinaria attached to the palps of a *M. brasiliensis* moth. G) *H. paulistana* pollinaria attached to the posterior lower margin of *Eumorpha obliquus* eye after manually contacting the hawkmoth head to the floral column. Scale bar equals 1 cm.

The orchid species studied are terrestrial herbs growing generally in grasslands with swampy soils and on the margins of streams and ponds. *H. gourlieana* grows in Northern Argentina, Bolivia, Uruguay, and Central to Southern Brazil, *H. johannensis* grows in Brazil, Paraguay and Bolivia, and *H. paulistana* is endemic to the Atlantic Rainforest of the São Paulo State in South-eastern Brazil [33]. The three species have long inflorescences with a variable number of large flowers (3 up to 30), which are greenish-white in colour and emit pleasant scents after dusk [32], [33]. Flower architecture is bilabiate, with an upper lip formed by the median sepal and lateral petals that cover the column, and a lower lip formed by the dissected labellum. Posteriorly the labellum develops into a very long and slender spur (up to 170 mm long) in which nectar is accumulated (Figure 1A). Spurs are partially or completely enclosed by the bracts of the flowers located below. The rostellum bears two lateral stalks that present the viscidia apically. Rostellum morphology differs among the three species leading to pollinaria attachment on different areas of the hawkmoths’ heads: the anterior margin of the eyes in *H. gourlieana* (Figure 1 B, E) [32]; the labial palps in *H. johannensis* (Figure 1 C, F; F.W. Amorim, G.E. Wyatt, M. Sazima unpublished data); and the posterior lower margin of the eyes in *H. paulistana* (Figure 1 D, G; F.W. Amorim, G.E. Wyatt, M. Sazima unpublished data). Flowers bear two long stigmatic stalks located at either side of the spur entrance and below the viscidia (Figure 1 B-D). Fruit set is pollinator-dependent in these three *Habenaria* species, as flower morphology precludes self-pollination (F.W. Amorim, G.E. Wyatt, M. Sazima unpublished data) [32].

Fieldwork was carried out in a population of *H. gourlieana* located in El Durazno, Córdoba province, Argentina (31°21’S, 64°36’W, 1200 m a.s.l.) during the flowering seasons 2004 and 2005. The studied populations of *H. johannensis* and *H. paulistana* were located in the Núcleo Santa Virgínia (NSV) at Serra do Mar State Park, São Paulo State, Brazil. At this site *Habenaria johannensis* occurs exclusively along the road Oswaldo Cruz that traverses NSV (23°22’S, 45°11’W, 900 m a.s.l.). The *Habenaria paulistana* population is located inside the NSV (23°19’S, 45°08’ W, 914 m a.s.l.). Observations were made during the 2010-2011 flowering seasons of *H. johannensis* and during 2009-2011 for *H. paulistana*. The populations of the two *Habenaria* species studied in the area of NSV do not overlap in either flowering phenology or spatial distribution, and occur in sites with distinct microclimatic conditions generated by Serra do Mar mountain chain.

### Pollinators

Hawkmoths were collected using light traps (16 hours in the *H. gourlieana* population, 72 hours in the *H. johannensis* population and 144 hours in the *H. paulistana* population). We used different time periods to collect hawkmoths because in the Brazilian sites the hawkmoth assemblage was more diverse so that an extra effort was necessary to sample the hawkmoth species present in the area during orchid flowering periods [29], [31]. Proboscis lengths were measured in captured hawkmoths using digital calipers (error, 0.01 mm) and moths were carefully inspected under a binocular microscope to determine if they were carrying orchid pollinaria or pollen loads from other plant species. In order to track actual pollinators (moths carrying pollinaria), we performed additional nocturnal observations of pollinator visits to flowering plants totalling 10 hours for *H. gourlieana*, 12 hours for *H. johannensis* and 80 hours for *H. paulistana*.

Because we did not record any natural pollinator visits to *H. johannensis* flowers during field observations, we attempted in February 2011 to test experimentally the ability of hawkmoths to pollinate this plant with a flight cage of 2 m^3^ mounted over a flowering plant. Ten hawkmoths of five species (two *Agrius cingulata*, one *Adhemarius eurysthenes*, two *Manduca diffissa*, two *M. florestan*, two *M. brasiliensis* and one *Xylophanes crenulata*) were captured in a light trap, and released within the cage, and their activity was observed under starlight conditions with the aid of night vision goggles (*Eyeclops*®) over three consecutive nights.

### Traits Measured

We measured spur lengths using digital calipers (error, 0.01 mm). To avoid serial variation along the inflorescence we only measured the first freshly open flower in the inflorescence. To determine the minimal tongue length required to reach the nectar in the flower spur, we also measured the nectar column height in a sample of 20 flowers from 6 to 10 different plants of each species. Flowers were covered for a period of 48 hours in order to prevent pollinator access and to allow nectar to accumulate in the spur. We also recorded the total number of flowers produced by each plant during the flowering season as well as plant height, as these variables may affect pollinator attraction and reproductive success [17], [22], [34]. However, since both variables were significantly correlated, subsequent analyses were performed using only the number of flowers.

To estimate reproductive successes, we recorded at the end of flowering season the number of fruits (female reproductive success) and the number of removed pollinaria (male reproductive success) per plant. The latter was possible since unremoved pollinaria remain intact in wilted flowers and developing fruits. Reproductive success was recorded for 81 *H. gourlieana* plants in 2005, 64 *H. paulistana* in 2010 and 63 *H. johannensis* in 2011.

### Estimating Selection Measures and Phenotypic Selection Analyses

We compared the reproductive success of the three *Habenaria* species using generalized linear models with binomial or Poisson error structure and penalized quasi-likelihood to control over-dispersion [35]. Before conducting phenotypic analyses, the fitness values of each plant were divided by the corresponding population mean and plant traits were standardized. The intensity and pattern of phenotypic selection acting on spur length, and flower number, were estimated using the methodology of Lande & Arnold [36]. Separate phenotypic selection analyses were undertaken for each species as well as fitness measures. Selection gradients were estimated by multiple regressions to evaluate the direction and magnitude of selection on a specific trait independent of the indirect effect of other traits. Significant linear gradients (β) indicate that selection favours either higher (if positive) or lower (if negative) trait values, inducing changes in population means. Significant nonlinear selection gradients (γ) indicate convex nonlinear selection against extreme phenotypes (stabilizing selection), concave nonlinear selection against intermediate trait values (disruptive selection), or correlational selection on a given combination of traits.

Because residuals from regression analyses departed from normality, standard errors for selection gradients were calculated using bootstrap methods [37]-[39]. We generated 10,000 bootstrap samples from the original data set. Selection gradients estimated after each bootstrap were used to obtain a frequency distribution. A selection gradient was considered significant if the bias-corrected confidence percentile interval did not include zero [37]-[39]. We used the *boot* [40] package of R software vs. 2.13.0 [41] to perform the bootstrapping and to estimate the 95%, 99% and 99.9% confidence intervals.

Because multiple regressions are constrained to adjust the best linear or quadratic approximation to the fitness surface, direct interpretation of selection gradients may occasionally be misleading [42], [43]. Hence, we applied the cubic spline non-parametric regression to avoid *a priori* assumptions about the shape of the relationship between traits and fitness [44]. We used the *gam* routine of *mgcv* package [45] of R software vs. 2.13.0 [45] to estimate the cubic splines. For each univariate spline, we fixed the covariate at its mean value. Smoothing parameters were obtained by minimizing the generalized cross-validation scores [46], and Bayesian standard errors were obtained according to Wood [45].

## Results

### Pollinators

**Figure 2:**
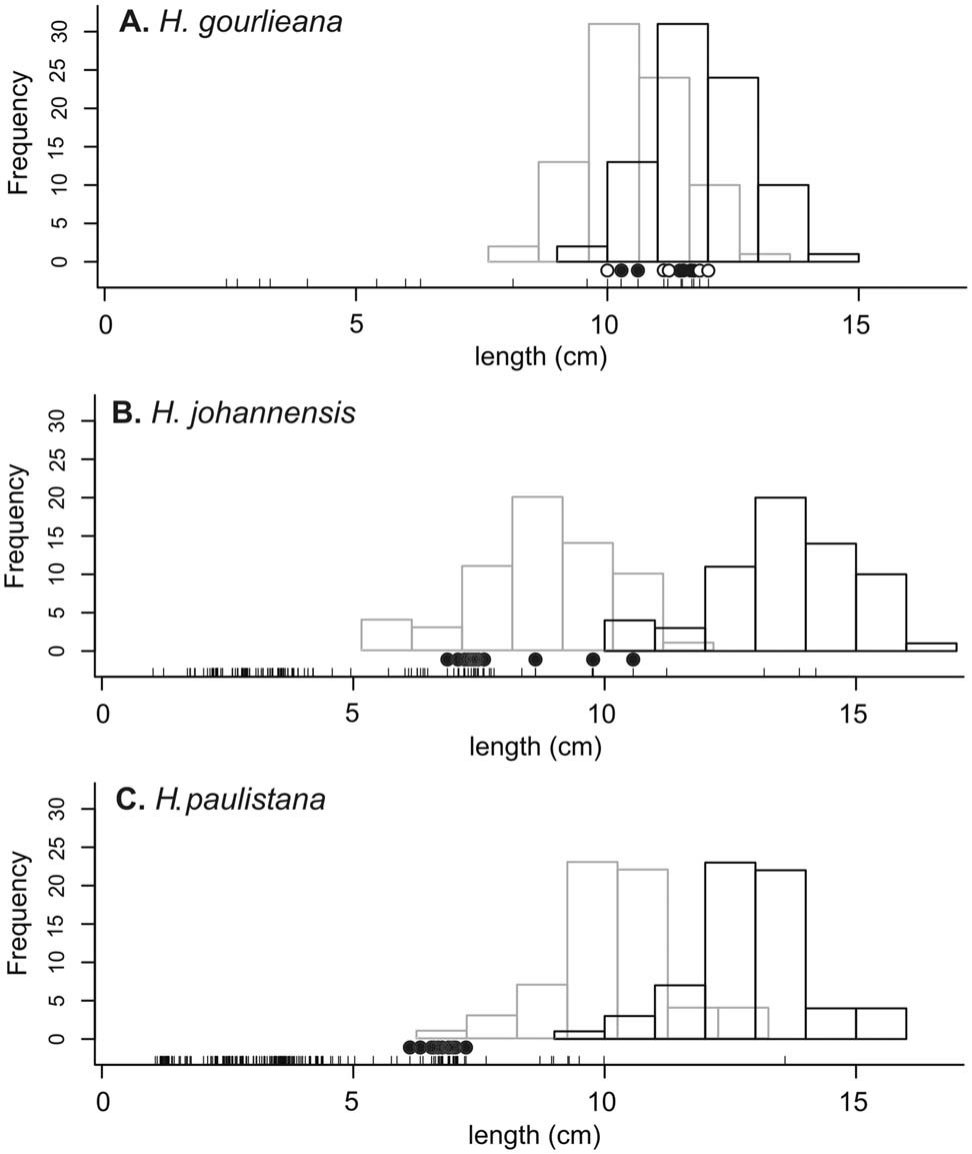
Match and mismatch between the lengths of orchid spurs and pollinators’ proboscises. Black bars show spur length distributions in the three *Habenaria* species and grey bars the corrected histograms according to the mean height of the nectar column within the spur. The vertical black lines in the *x* axis show the proboscis lengths of all captured hawkmoths, circles represent individuals from those species seen either visiting flowers (filled) or captured carrying pollinaria attached to their eyes (open).

As is commonly found in the Neotropical region, the studied hawkmoth communities mainly comprised short-tongued moths and a minority of long-tongued ones (Table 1). The three *Habenaria* species were, however, pollinated exclusively by those few hawkmoth species with the longest proboscises, which were potentially able to reach the nectar concealed in the very long spurs (Figure 2, Table 1). The nectar column filled up to a third of the flower spurs (*H. gourlieana* 27.65±11.69 mm, *H. johannensis* 47.06±9.55 mm and *H. paulistana* 34.87±8.89 mm). Thus, recorded pollinators were able to access nectar in almost all *H. gourlieana* and *H. johannensis* flowers (Figure 2A, B), but only in the shortest flowers of *H. paulistana* (Figure 2C).

**Table 1:** Hawkmoth species captured during flowering season in the studied populations.

Hawkmoth species	Mean proboscis length ± SD (n)
***Habenaria gourlieana*** ** population**	
*Manduca sexta* (Linnaeus, 1763)*	109.61±10.93 (12)
*Agrius cingulata* (Fabricius, 1775)	95.96 (1)
*Manduca diffissa* (Butler, 1871)	58.94±4.44 (3)
*Erinnyis oenotrus* (Cramer, 1780)	40.34 (1)
*Erinnyis ello* (Linnaeus, 1758)	30.83 (1)
*Xylophanes tersa* (Linnaeus, 1771)	28.58±6.15 (2)
*Erinnyis obscura* (Fabricius, 1775)	26.39 (1)
***Habenaria johannensis*** ** and ** ***H. paulistana*** ** populations**	
*Manduca rustica* (Fabricius, 1775)	132.86±10.17 (3)
*Manduca janira* (Jordan, 1911)	101.61±9.38 (3)
*Agrius cingulata* (Fabricius, 1775)***	95.65±8.18 (4)
*Cocytius duponchel* Poey, 1832	84.27 (1)
*Manduca brasiliensis* (Jordan, 1911)**	71.48±7.44 (22)
*Manduca diffissa* (Butler, 1871)	71.44±3.45 (18)
*Manduca florestan* (Stoll, 1782)	61.28±4.93 (10)
*Manduca lefeburii* (Guérin-Méneville, 1844)	53.99 (1)
*Eumorpha analis* (Rothschild & Jordan, 1903)	49.19±8.02 (5)
*Pachylia fícus* (Linnaeus, 1758)	48.33±3.32 (5)
*Pseudosphinx tetrio* (Linnaeus, 1771)	47.69 (1)
*Xylophanes chiron* (Drury, 1773)	47.07±3.85 (5)
*Eumorpha obliquus* (Rothschild & Jordan, 1903)	44.54±0.94 (2)
*Erinnyis alope* (Drury, 1773)	43.37±2.69 (2)
*Eumorpha translineatus* (Rothschild, 1895)	43.18±2.22 (10)
*Erinnyis oenotrus* (Cramer, 1780)	39.78±3.50 (10)
*Xylophanes titana* (Druce, 1878)	36.71±2.42 (10)
*Erinnyis ello* (Linnaeus, 1758)	36.11±1.88 (24)
*Xylophanes crenulata* (Vaglia & Haxaire, 2009)	36.01±2.08 (14)
*Xylophanes indistincta* Closs, 1915	34.4 (1)
*Xylophanes aglaor* (Boisduval, 1875)	34.12±2.33 (5)
*Xylophanes xylobotes* (Burmeister, 1878)	34.10±1.40 (6)
*Xylophanes tersa* (Linnaeus, 1771)	32.86 (1)
*Xylophanes thyelia* (Linnaeus, 1758)	30.78±4.53 (11)
*Pachylioides resumens* (Walker, 1856)	26.70±2.30 (6)
*Adhemarius gannascus* (Stoll, 1790)	26.53±1.49 (4)
*Xylophanes isaon* (Boisduval, 1875)	26.10±3.49 (23)
*Adhemarius eurysthenes* (R. Felder, 1874)	26.03±1.47 (14)
*Enyo ocypete* (Linnaeus, 1758)	25.43 (1)
*Xylophanes porcus* (Hübner, 1823)	22.86±1.85 (17)
*Callionima nomius* (Walker, 1856)	18.50±0.08 (7)
*Callionima innuus* Rothschild & Jordan, 1903	16.60±0.95 (2)
*Nyceryx alophus* (Boisduval, 1875)	13.56±1.56 (5)
*Perigonia lusca* (Fabricius, 1777)	12.74 (1)
*Perigonia pallida* Rothschild & Jordan, 1903	12.15±0.84 (8)
*Orecta lycidas* (Boisduval, 1875)	10.18 (1)

Species are arranged in decreasing order according to their mean proboscis length (mm). Asterisks show species carrying *H. gourlieana* (*) and *H. paulistana* (**) pollinaria attached to their eyes, or *H. johannensis* (***) pollinaria attached to their palps. Nomenclature and classification generally follows Kitching *et al.* [60].

Seven hawkmoth species were recorded for the *H. gourlieana* population (Table 1), but only five individuals of *Manduca sexta* were observed carrying pollinaria attached to the fore margin of their eyes (Figure 1E, 2A). *M. sexta* individuals also carried pollen from other six plant species present in the community, two of them having flowers with nectaries as long as *H. gourlieana*: *Oenothera affinis* Cambess. (Onagraceae) and *Mandevilla petraea* (A. St.-Hil.) Pichon (Apocynaceae), two with relatively short corolla tubes, *Cestrum parqui* L’ Her. (Solanaceae), the exotic *Mirabilis jalapa* L. (Nyctaginaceae) and two unidentified pollen types.

A total of 36 hawkmoth species were recorded in the area during the flowering seasons of *H. johannensis* and *H. paulistana* (Table 1). None of the light-trapped moths were observed carrying pollinaria and we did not record any visits in the field to *H. johannensis* flowers. But in the flight cage experiment, two of the five hawkmoths species released inside the cage visited *H. johannensis* flowers: *A. cingulata* (n = 2) and *M. brasiliensis* (n = 2), of which only *A. cingulata* removed pollinaria in one of the visits (Figure 2B). Pollinaria of *H. johannensis* were attached to the palps of the *A. cingulata* individual. We recorded six hawkmoth visits to *H. paulistana* (Figure 2C), one from the long-tongued *M. brasiliensis.* The remaining visits were identified to genus level: a long-tongued *Manduca* (n = 4) and a short-tongued *Xylophanes* (n = 1). Putative hawkmoth pollinators of *Habenaria* species in Brazil were carrying pollen from other plants present in the community, mainly from the very long-tubed flowers of *Posoqueria latifolia* (Rudge) Roem. & Schult. (Rubiaceae) and *Hillia parasitica* Jacq. (Rubiaceae), as well as the brush-type flower of the legume *Inga sessilis*.

**Figure 3:**
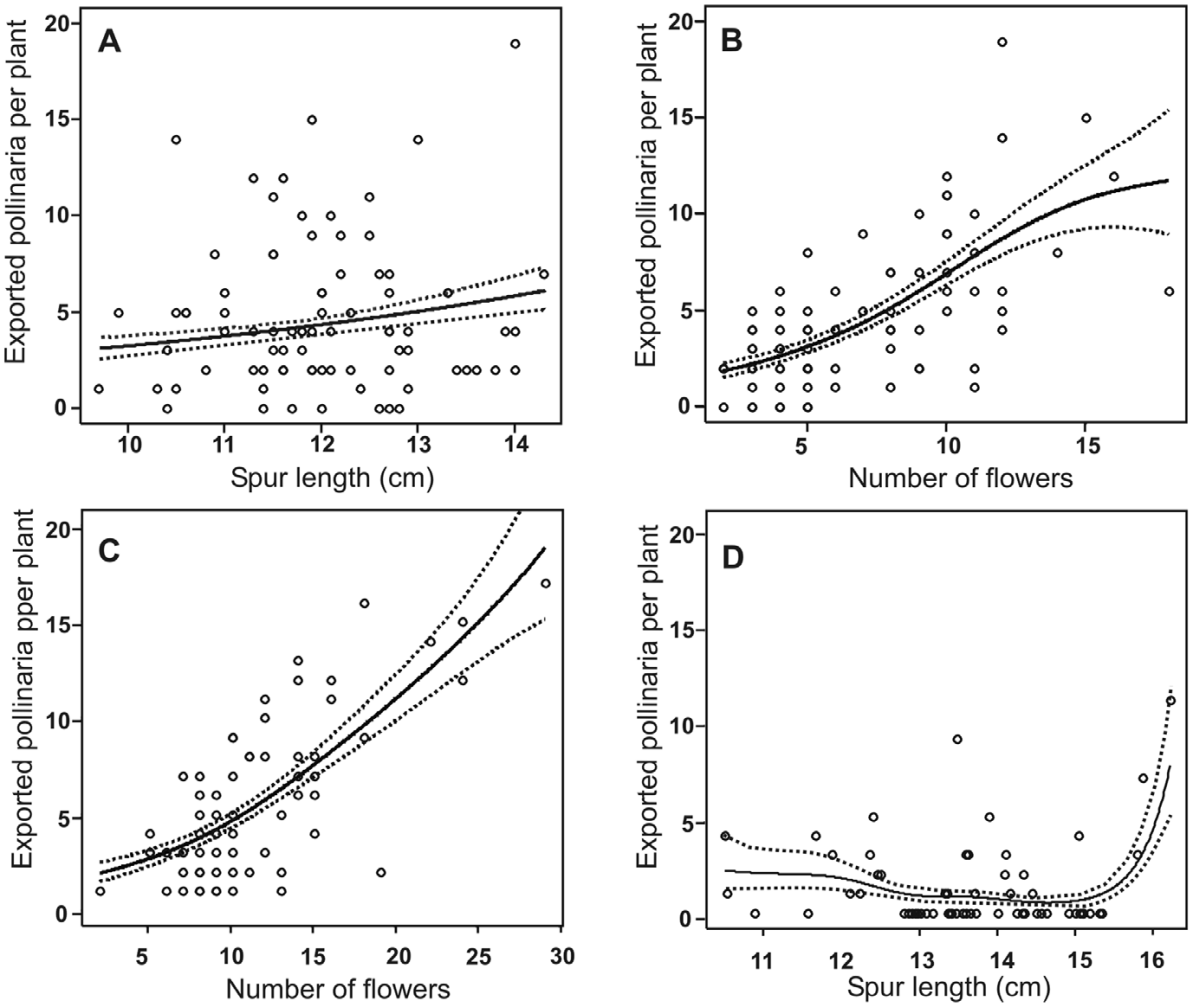
Cubic spline regressions between floral traits (spur length and number of flowers) and reproductive success (exported pollinaria) in three orchid species. *Habenaria gourlieana*: A & B. *H. paulistana*: C. *H. johannensis*: D. Dotted lines show ±1 Bayesian standard errors.

### Phenotypic Selection Analyses

*Habenaria* species differed in fruit set (*F_2,205_* = 426.94, P<0.0001) and in the proportion of removed pollinaria (*F_2,205_* = 548.62, P<0.0001). In both cases *H. johannensis* achieved the lowest relative success (Table 2). Regarding total fitness, *Habenaria* species differed in the number of fruits produced per plant (*F_2,205_* = 239.23, P<0.0001) and in the number of exported pollinaria (*F_2,205_* = 199.17, P<0.0001). Again, *H. johannensis* had the lowest total reproductive success (Table 2).

**Table 2:** Mean and standard deviations of reproductive success estimates in three species of *Habenaria*.

Reproductive success measure	Mean (SD)		
	*H. gourlieana* (n = 81)	*H. johannensis* (n = 63)	*H. paulistana* (n = 64)
Fruits/flowers	0.395 (0.323)	0.036 (0.068)	0.288 (0.289)
Pollinaria exported/flowers	0.246 (0.128)	0.048 (0.072)	0.364 (0.216)
Fruits per plant	4.734 (4.958)	0.571 (1.266)	2.284 (2.753)
Pollinaria exported per plant	5.703 (4.173)	1.365 (2.253)	4.765 (3.909)

Significant directional selection acting on spur length was observed only through male function (number of exported pollinaria per plant) in *H. gourlieana* (Table 3, Fig. 3A), although, a significant disruptive selection gradient through male function was detected in *H. johannensis*. This pattern, however, was not evident in cubic splines because only a small group of long-spurred plants attained high reproductive success (Table 3, Fig. 3F). Significant directional selection acting on flower number through male function was observed in *H. gourlieana* and *H. paulistana* (Table 3, Figs. 3B, 3D). In addition, significant directional selection was detected for flower number through female function (number of fruits) in *H. gourlieana* and *H. paulistana* (Table 3, Figs. 3C, 3E).

**Table 3:** Multivariate phenotypic selection on spur length and flower number through male (number of exported polinaria per plant) and female (number of fruits) functions in *Habenaria gourlieana*, *H. johannensis* and *H. paulistana.*

Species	Character	Male function				Female function			
		β_i_ (SE)		γ_ii_ or γ_ij_ (SE)		β_i_ (SE)		γ_ii_ or γ_ij_ (SE)	
*Habenaria gourlieana* (n = 81)	Spur length	0.153 (0.083)	*	0.015 (0.108)		0.139 (0.108)		0.124 (0.170)	
	Flower number	0.563 (0.088)	***	0.083 (0.173)		0.801 (0.111)	***	0.430 (0.189)	*
	Spur length × flower number			0.225 (0.112)				0.126 (0.141)	
*Habenaria johannensis* (n = 63)	Spur length	0.027 (0.305)		1.219 (0.524)	**	0.185 (0.260)		0.434 (0.512)	
	Flower number	0.296 (0.259)		0.521 (0.489)		0.572 (0.472)		0.796 (0.894)	
	Spur length × flower number			-0.389 (0.359)				-0.364 (0.470)	
*Habenaria paulistana* (n = 64)	Spur length	0.114 (0.073)		-0.137 (0.124)		0.138 (0.119)		-0.219 (0.177)	
	Flower number	0.484 (0.074)	***	-0.039 (0.132)		0.600 (0.178)	***	0.305 (0.274)	
	Spur length × flower number			0.144 (0.129)				-0.011 (0.232)	

Standardized linear selection gradients (β_i_), non-linear selection gradients (γ_ii_), correlational selection gradients (γ_ij_) and standard errors (SE) are shown. Standard errors and significance of selection gradients was estimated using 10,000 bootstrap samples and testing if bias-corrected accelerated percentile intervals include zero. *P<0.05; **P<0.01; ***P<0.001.

## Discussion

The three studied *Habenaria* species share very precise pollination mechanism in which pollinaria are attached to the hawkmoths’ eyes or labial palps when they introduce the proboscises to drink the nectar accumulated in the very long spurs (Fig. 1B-D). Putative pollinators of these species belonged to those few hawkmoth species with the longest proboscises in the two studied communities (Table 1). Although spur length was similar among species, only *H. gourlieana* had a close match with the local long-tongued hawkmoth fauna. Most of the pollinators of this species (those moths bearing pollinaria attached to their eyes) had proboscises long enough to completely deplete or at least to have access to some nectar in nearly every flower in the population (Fig. 2). In the populations of the other *Habenaria* species spur length did not match the proboscises of the local fauna, with the exception of some few long-tongued hawkmoths able to reach nectar. This mismatch was somewhat compensated in *H. johannensis* by a relatively higher nectar column, and this permitted a closer nectar-proboscis match that could allow most pollinators to access nectar in approximately half of the flowers; a few pollinators could access nectar even from the longest flowers, although they were not able to completely deplete them. In *H. paulistana* the nectar-proboscis mismatch was so strong that only a very small fraction of the flowers had nectar accessible to the putative pollinators (Fig. 2).

*Habenaria* species differed in male and female efficiency (Table 2). Few flowers set fruits or achieved pollinaria exportation in *H. johannensis*, despite the marked hawkmoth activity recorded for other plant species of the community that offer large amounts of nectar, such as *Inga sessilis* [47]. These observations suggest that hawkmoths learn to avoid a species which flowers offer little accessible nectar [48] and, consequently, the population is on average not sufficiently rewarding. *Habenaria gourlieana* and *H. paulistana* had comparatively high reproductive success but this did not always mean high pollen transfer efficiency and, ultimately a high reproductive output in the populations of these species. In fact, frequent visitation rates in *H. gourlieana*, indicated by the number of light-trapped moths carrying pollinaria and by direct observations in the field, accounts for higher pollinaria removal but this does not translate in higher fruit set, revealing a low efficiency in pollen transference. Further studies are needed to determine whether the more precise mechanism of pollinaria deposition in *H. paulistana*, which has rigid pollinaria caudicles, could account for the higher pollen transference efficiency, as indicated by low pollinaria removal, but high fruit set.

Directional selection was only detected in favour of longer spurs in the *H. gourlieana* population and through male fitness (Table 3, Fig. 3A). The flower adaptive optimum was beyond the actual spur length mean because pollinators of this species had proboscis lengths that equalled or exceeded the spur length mean [49], and so only plants with the very longest spurs compel hawkmoths to contact their eyes with the viscidia while introducing proboscis to reach the concealed nectar. A similar pattern was observed in previous pollinator-mediated selection studies performed on other orchid species [15]-[17]. In contrast, we did not find directional selection on spur length in *H. johannensis* or in *H. paulistana* populations (Table 3). Although a significant disruptive gradient was observed in *H. johannensis*, cubic spline analysis showed that most plants with spur lengths around the mean attained extremely low reproductive success and three long-spurred individuals with high fitness were responsible for most of the observed fecundity (Fig. 3D). Most pollinators of this species had proboscises shorter than the mean spur length and those few individuals with proboscises long enough to reach the nectar (*e.g. Manduca rustica*) were not observed visiting flowers or carrying pollinaria (Table 1). Although spur length varied from 98 to 159 mm in *H. paulistana*, most flowers were functionally similar because hawkmoths cannot obtain reward from them. Hence, due to this armament imbalance in plant-pollinator traits, no selection favouring longer spur phenotypes was found.

The mismatch apparently explains the absence of selection on spur length and poses the question of why did these orchid species develop such extremely long spurs? As a general pattern, plants usually have more exaggerated morphological traits than pollinators, because of the intrinsic differences in their physiological and developmental constraints [9]. Moreover, Anderson *et al*. [9] also noted that selection strength in floral and pollinator traits is dependent on the community context. Since putative pollinators of these three orchids also visit many other plant species in their respective communities, diffuse evolution processes may ensure that trait match selection in a particular plant species is less likely to occur [9], [50].

Furthermore, the absence of moths with very long proboscises which could act as selective agents may be attributed to spatiotemporal fluctuations in the hawkmoth assemblage [31], [51]. It is known that insects of this group are prone to fluctuations in their abundance due to changes in climatic conditions, as well as resource availability for their larvae [31], [52]-[54]. It is possible, therefore, that selection on spur length in these orchids may have operated in a period in which hawkmoths with long proboscises were more abundant. Thus, the current spur length may be the result of punctuated selection events through time [55]-[57]. Additionally, the response to such punctuated selection events would be delayed by the perennial life cycle and clonal reproduction of these orchid species, as less successful individuals can nevertheless remain in the population for many years [58]. Our findings may be relevant in the current scenario of global climate change, a factor that could affect phenology, abundance and distributions of plants and pollinators, generating temporal mismatches between mutualistic partners [25], [59]. In particular, *Habenaria paulistana*, an endemic species with a very restricted distribution in the Atlantic Rainforest [33], may face a progressive absence of pollinators (due to spatial and phenological mismatches), and therefore a lack of sexual reproduction.

We detected phenotypic selection consistent with the functional match between flower morphology and pollinators as would be expected since Darwin’s classical work on hawkmoth-pollinated orchids. However, our findings also indicate that pollinator-mediated selection could be a punctuated mechanism that varies through time and space [56]-[57]. In conclusion, this study highlights the need to consider local variations in pollinator assemblages across landscapes and their impact in moulding flower morphology.
